# Complementing, competing, or co-operating? Exploring newspapers’ portrayals of the European Parliament and national parliaments in EU affairs

**DOI:** 10.1080/07036337.2017.1281262

**Published:** 2017-01-31

**Authors:** Olga Eisele

**Affiliations:** ^a^ Department of Political Science, Institute for Advanced Studies, Vienna, Austria

**Keywords:** European Parliament, European Union, legitimacy, media, national parliaments

## Abstract

The paper explores newspapers’ portrayals of the European Parliament and national parliaments (NPs) in European Union (EU) affairs. To understand underlying perceptions of journalists, it takes public parliamentary activities and looks at their influence on parliaments’ news visibility in Finland, Germany and the UK in routine periods in 2011 and 2012. This is done against the background that parliaments, regarded as ultimate legitimisers of state power, depend on the mass media to reach their citizenry. However, journalists follow their own agenda in publishing parliamentary news. In this regard, they may highlight the complementarity, competition or cooperation of parliaments in the EU’s unique multi-tier environment. Overall, our results suggest that NPs correspond stronger with newsmakers’ anticipation of readership interest. In addition, findings seem to support the assumption that parliaments in the EU are mostly perceived as complementary, separate legislative branches in EU decision-making.

## Parliamentary communication in the European Union

Parliamentary representation is still regarded the ultimate legitimiser of state power (Urbinati and Warren 2008, 390). For a polity like the European Union (EU) which is continuously diagnosed to suffer from a severe democratic deficit (Follesdal and Hix 2006; Hooghe and Marks 2009), the link between voters and their representatives is particularly crucial for the legitimacy of political decisions: Citizens need to know how the representatives they have directly elected make their interests count (e.g. Manin 1997). How can they otherwise judge if the EU’s policy output is legitimate – i.e. worthy of being accepted? To reach citizens, parliaments need to be visible in public. In their efforts to do so, they are to a great extent depending on the mass media as a channel to reach a broad audience. Media set the agenda of the information environment in which citizens form their opinions, and they may do so differently in different societies (Blumler and Kavanagh 1999; Habermas 2012, 136; Hallin and Mancini 2004). Generally speaking, news items produced by parliaments have to pass a certain threshold that is defined by the newsworthiness of these activities and the respective parliament itself (e.g. O’Neill and Harcup 2009). It is for this reason that ‘[European] integration [scholars] need to understand how the media act as selective amplifiers of political information about the EU and how selected outputs are turned into news that shape the political reality of Europe’ (Trenz 2008, 15). Therefore, the broader aim of this study is to investigate how well parliaments in the EU succeed in receiving attention from the national media.

The EU represents a unique ‘laboratory’ (Bellamy 2006) case in this respect: In its complex system of multilevel governance, authority is dispersed across different levels. Together with the European Parliament (EP), national parliaments (NPs) find themselves in an indeterminate field of political representation in which they co-exist influencing each other and representing overlapping constituencies without a clearly defined relationship. Parliamentarians at both levels are directly elected agents of the citizens but have very different tasks in the legislative process of the EU. Accordingly, the academic discussion on interparliamentary relationships has highlighted its competitive, co-operative and complementary facets (e.g. Crum and Fossum 2009; Maurer 2009; Wouters and Raube 2012).

European policies are dealt with by both parliaments and therefore are the area in which interparliamentary problems may become most explicit. Against this background, the question investigated in this paper is the following: *How do parliaments in the EU influence each other with regard to their news visibility in EU affairs?* The literature has, to the best of knowledge, not addressed this topic to date: Studies tend to either deal with parliaments in the media without connecting the two levels (e.g. Vos 2015) or with interparliamentary relations without considering communication about them (e.g. Crum and Fossum 2013; Herranz-Surrallés 2014). To tackle this research gap, the paper takes an innovative approach and for the first time brings together data on the communication by and about both parliamentary levels. It takes debates, oral and written questions and press releases of the EP and NPs in EU affairs as proxies of parliaments’ efforts to become visible and explores their effects on newspaper coverage in routine periods in 2011 and 2012. The sample consists of articles in the three largest newspapers in Finland, Germany and the United Kingdom dealing with the EP or NPs in EU affairs.

## Parliaments and EU politics in the media – a framework for analysis

To develop a framework for the comparative analysis of parliaments’ general visibility in the news, we will first take a closer look at newsmakers’ motivations to select news items for publication in general. On that basis, we will then trace the general discussion on the multilevel system of parliamentary representation highlighting the complementary, competitive and co-operative nature of parliamentary interaction. We assume that these patterns of interaction may underlie journalists’ perceptions when evaluating parliaments as news items and then formulate ideal-type hypotheses to explore effects of activities on coverage.

### Newsmakers’ selection criteria

Not parliaments themselves, but newsmakers decide on what becomes news: parliaments and their activities undergo a selection procedure marked by standards of relevance or newsworthiness and the constraints of the news production machinery. As Tuchman (2002) sums up, news organisations are situated in a field of competing forces including the news market, politics creating the legal framework of news, newsmakers’ sources as informants but also lobbyists for visibility at the same time, and last but not least, their audience. The audience’s approval and loyalty is essential for any news organisation’s survival and hence, the anticipation of readership interest, i.e. as mirrored in public opinion, is central. Audiences, here, comprise not only citizens as readership but also other journalists evaluating the quality of news produced by their peers (Tuchman 2002, 80). Hence, journalists’ work is constrained by accessibility of sources and market considerations, such as limitations of space, while they need to remain credible at the same time (see also Shoemaker and Reese 1996; Sparrow 2006).

Keeping these considerations in mind, scholars have tried to abstract the decisive factors in the news selection process. On the one hand, journalists are seen as chroniclers of what is happening (McQuail 1992) and accordingly, are expected to ‘give an accurate account of important events, actions, and interventions within the institutionalized arenas of the political system and make the political process transparent for the citizen public’ (Tresch 2009, 70). In a similar vein, the media has been described as a watchdog or even a fourth estate with the task to ‘spotlight and draw public attention to problems and situations that need solutions and repair’ (Shoemaker 2006, 108; see McNair 2009 for an overview).

On the other hand, the ‘importance’ of events, actions and interventions is evaluated by virtue of their newsworthiness which in itself is a slippery concept that cannot serve to predict news (Shoemaker 2006, 105). There seems to be agreement among professionals regarding when news is worthy of being published (O’Neill and Harcup 2009; also Shoemaker 2006); but the actual selection procedure has been described as being subjective, based on journalists’ cognitive predispositions (Donsbach 2004) and depending on circumstances rather than objective standards. Ultimately, this makes the concept of newsworthiness contingent and ‘one of the most opaque structures of meaning in modern society’ (Hall 1973, 181, quoted in O’Neill and Harcup 2009).

Accordingly, studies of news factors have mainly produced more or less extensive lists of the most common factors identified empirically and ex-post that have served to formulate expectations (Galtung and Ruge 1965; see O’Neill and Harcup 2009 for an extensive overview). As the most important, the relevance, salience or proximity of events, power or influence of involved actors, conflict, magnitude with regard to the amount of people affected by the news, and the negative consequences of an event have been listed.

Against this background, we will now turn to a short discussion of findings that the literature provides on parliaments in EU affairs. In doing this, we will seek to understand the ways in which parliamentary relationships may be perceived and accordingly, how journalists may make parliaments and their activities visible.

In general, the discussion regarding the ties between both parliamentary levels mainly evolves around the central question if, under what conditions and in what forms parliaments in the EU are interacting. It focusses mostly on theoretical issues (see Benz 2016 and Crum and Fossum 2009 and for detailed comparative discussions), (inter-)institutional dependencies or developments (e.g. Katz and Wessels 1999; Neunreither 2005), inter-parliamentary cooperation (e.g. Crum and Fossum 2013) and inter-parliamentary competition (e.g. Herranz-Surrallés 2014).

### The EP and NPs as complementary legislative branches in EU politics

Some studies elaborate on how the EP and NPs complement each other in EU politics in a sense of dividing tasks between different parliamentary branches (e.g. Maurer 2009). Looking into Common Security and Defence Policy (CSDP), Wouters and Raube (2012), for example, point out that the EP has greater possibilities to investigate cross sectional policy issues (Ibid., 160) since it can access information touching upon different supranational policies more easily at EU level; NPs, in turn, decide over the national defence budget and can hold national executive actors accountable in CSDP more effectively. Also regarding other EU policies, the EP and NPs fulfil very different tasks and therefore usually become active at different points in the EU’s legislative cycle. Therefore ‘it is important that parliaments understand that the EP and national parliaments complement each other’ (Wouters and Raube 2012, 160; also Maurer 2009). Journalists would then be expected to portray parliaments and their activities independently from each other.

In this respect, earlier research has found that parliamentary coverage has generally decreased because parliaments’ work is often perceived as too predictable on the one hand and too complex on the other hand to meet the readership’s taste (Koopmans and Statham 2010; Negrine 1999). There is an abundance of studies focusing on the EU in general taking EP elections as the period of investigation (instead of many: Boomgaarden et al. 2013) or on national elections, NPs or national politics more generally (for an overview see Vos 2015). However, the EP in the media has rarely been discussed (but see Gattermann 2011, 2013; Gattermann and Vasilopoulou 2015); the same is true for news about NPs in EU affairs (but see Auel, Eisele, and Kinski 2015). Notwithstanding their decreased general newsworthiness, however, parliaments are still relevant and powerful institutions that take legally-binding decisions: ‘Because their actions directly affect many citizens, state actors actually benefit from an “inherent” news value and get more access to the media’ (Tresch 2009, 71). In addition, the EU has been found to be increasingly salient and covered more during the financial crisis due to the increased conflictuality of the topic (Michailidou and Trenz 2010). Also regarding domestic parliamentary activities, NPs are found to mirror the increased interest in EU affairs and engage in public debate to a greater extent (e.g. Auel, Eisele, and Kinski 2016; Auel and Raunio 2014).

Accordingly, empirical studies focussing on the mirroring of individual political actors’ activities in the news and parliamentary activities as such (Auel, Eisele, and Kinski 2015; Van Santen, Helfer, and van Aelst 2015) have found a positive influence of greater public visibility on coverage. Some studies have also suggested a more ambiguous influence (e.g. Gattermann and Vasilopoulou 2015), but there is evidence lending support to the expectation that more efforts to become visible are rewarded by newsmakers (see Vos 2015 for an extensive overview). Summing up, assuming that newsmakers perceive parliaments as independent links in the EU’s legislative chain, parliaments can, independently from each other, be expected to increase coverage with their activities.

*H*_*1-A*_ *:* The more active the European Parliament is, the more visible it is in press coverage.

*H*_*1-B*_ *:* The more active the national parliament is in EU affairs, the more visible it is in press coverage.

### EP and NPs as direct competitors or co-operators in EU politics

Regarding a direct comparison of parliaments, Crum states that ‘the European Parliament … has come to style itself more and more on the example of national parliaments’ (2005, 453). Gattermann finds this statement reflected in journalists’ portrayals of the EP. She concludes that the EU’s parliament in the media is usually depicted in a manner facilitating (domestic) audience understanding, namely by presenting it as a parliament that follows the national tradition:

It is due to the distinct experience with the national parliamentary tradition that correspondents have difficulties in presenting the EP in a way that is understandable for their readers. The EP is different from its national counterparts. Correspondents are aware of that and do in fact draw comparisons to the national parliamentary culture to make it more perceptible for their readers (2011, 219).

Thus, the national parliamentary culture is found to act as journalists’ implicit proxy to explain the EP. However, these findings are not conclusive about what kind of effects could be expected. In the following, we formulate hypotheses based on the assumption that, if newsmakers perceive both parliamentary levels in a competitive or co-operative way, activities could cause mutual effects: Activities of one parliament would, positively or negatively, influence the visibility of the other.^1^ Acknowledging the different tasks and functions that parliaments at both levels fulfil (e.g. Wouters and Raube 2012), however, we formulate these hypotheses not as contradictory to H_1_ but rather perceive of mutual effects as potentially occurring in addition to independent ones.

#### Parliaments as co-operators?

On the one hand, contributions on interparliamentary relationships highlight their co-operative nature. Looking at both, horizontal (amongst NPs) and vertical (EP-NPs) interaction, there is often a positive normative tenor that emphasises synergy effects of interparliamentary co-operation concerning policy influence, the reduction of information asymmetries, the exchange of best practices, expertise and an enhancement of parliamentary power balancing the executive in the EU (Crum and Fossum 2009; Raunio 2000). With regard to the particular aspect of the visibility of the EU’s parliaments’ relationships, some contributions stress the possible enhancement of mutual understanding and stronger prominence of Europe in general by interparliamentary cooperation (Kraft-Kasack 2008; Wagner 2013).

In fact, there are many different forms of vertical interparliamentary co-operation in the EU ranging from formalised interparliamentary networks such as the Conference of the European Affairs Committees of NPs and the EP to informal visits of parliamentary delegations or speakers. In addition, in some countries, MEPs regularly engage in committee meetings or even the plenary of their NP; in turn, most NPs have own offices in the EP or close by (see Hefftler and Gattermann 2015 for an extensive overview). Some political groups organise their own conferences at EU level and the Green parties have been found to even closely co-ordinate their policy positions (Miklin 2013).

Against this general background, also journalists may perceive both parliamentary levels as an intertwined network of relevant actors, e.g. as equally informed, non-executive experts on EU topics that can be asked for their opinions, no matter at which level EU politics was debated. Concerning mutual effects, then, activities could increase attention for the other parliament as well; thus, in contrast to H_1_ formulated above, we would assume the occurrence of mutual *positive* effects, either alone or in addition to independent effects of activities on coverage of the respective active parliament. Accordingly, the ideal-type expectation would be that:

*H*_*2*_ *:* The more active one parliament (in EU affairs), the more visible the other is in newspaper coverage.

#### Parliaments as competitors?

On the other hand, however, scholars have pointed out the potential for competition in the relationship of the EP and NPs and have given empirical accounts of its emergence (Neunreither 2005; also Winzen, Roederer-Rynning, and Schimmelfennig 2014): NPs might see their own powers and capabilities – and ultimately the sovereignty of the nation state – undermined by the EP or also interparliamentary networks as such (Wouters and Raube 2012, 155). Illustrating this, Herranz-Surrallés finds that NPs are in opposition of a strong EP in Common Foreign and Security Policy (Herranz-Surrallés 2014); also Winzen, Roederer-Rynning, and Schimmelfennig (2014) suggest a similar dynamic with strong NPs opposing a (further) empowerment of the EP (see also Gattermann 2013). NPs’ fears to lose competencies are also connected to the perceived self-empowerment of the EP and the fact that governments felt the need to democratise the EU by strengthening the EP’s status (Hix and Høyland 2013; Rittberger 2005).

In accordance with this, journalists may perceive and picture the EP and NPs in a more competitive fashion. Regarding parliaments’ comparative newsworthiness, the NP may overtake the EP in terms of proximity or relevance for the (domestic) audience, also judging from the higher voter turnout in national elections (e.g. Hix and Marsh 2011). In a similar vein, the NP might be seen as a powerful and more effective scrutinizer of the national executive in EU politics (e.g. Auel, Rozenberg, and Tacea 2015a) making another parliament at EU level obsolete.

The EP, in turn, could be seen as an powerful legislative player on a par with other EU institutions that can influence EU legislation more directly (Gattermann 2011; 133). It may be perceived as stronger as and more influential than the NP in EU affairs on a larger scale (Crum and Fossum 2009, 253). Inferring that perceived more relevant activities of one parliament could push the other parliament in the background, the competitive nature of parliamentary relationships could be expressed in negative influences of activities on coverage of the other parliament. Again, in contrast to H_1_, we assume *negative* effects on coverage of the perceived rivalling parliament – alone or in addition to independent effects of activities on coverage of the respective active parliament. The ideal-type expectation in this case, then, would be that:

*H*_*3*_ *:* The more active one parliament (in EU affairs), the less visible the other parliament is in newspaper coverage.

## Methodology

The paper builds on a unique data base that was merged out of two sets from larger research contexts. The first set focusses on the EP in newspapers. The second one was created in the context of the PACE project^2^ concentrating on parliamentary communication of and press coverage about NPs in EU affairs.

Both datasets are based on an extensive content analysis of newspaper articles conducted by human coders, manually selected on the basis of terms describing (1) the EP, party groups in the EP and MEPs; and accordingly, (2) the domestic parliament, its party groups and MPs in connection with EU topics including the financial crisis. They were selected if they dealt with the EP or NPs in EU affairs to a substantial degree: If articles only mentioned parliaments very briefly (i.e. in a portrait of a politician, mentioning that he was an MEP 20 years ago), they were not included in the analysis since they did not present substantive parliamentary news. Furthermore, articles were not included if they were not published in the national issue: In Germany, for example, the tabloid contains sections that have changing contents depending on the area where they are distributed. All articles were retrieved from online databases, such as Lexis Nexis and Factiva, but also newspapers’ own databases (i.e. all Finnish newspapers).

The premise for the selection of Finland, Germany and the UK was to maximise diversity on public opinion variables that reflect the political climate in a country and thus present reference points for journalists’ evaluations of audience preferences. Moreover, the three countries differ in their electoral systems and parliamentary scrutiny strength in EU affairs (e.g. Hefftler et al. 2015).

As Western established democracies, countries do not differ much regarding their media systems (Hallin and Mancini 2004). However, they differ with respect to domestic parliamentary traditions. Such traditions influence the degree of publicity of parliamentary activity: The House of Commons as a classic debating parliament, for example, emphasises public debate whereas the Finnish Eduskunta as a working parliament stresses committee work which is usually not public. The Bundestag is categorised as somewhat of a hybrid, but is usually described as more of a working parliament (Steffani 1979; also Auel and Raunio 2014). The EP is described as a special case of a working parliament with controlling tasks rather than initiating ones (Dann 2003). Moreover, as discussed earlier, journalists are found to implicitly refer to domestic parliamentary traditions when explaining the EP to their readership at home (Gattermann 2011).

The study’s time frame comprises routine periods of 6 weeks in 2011 and 6 weeks in 2012 covering the last three weeks in May and the first three weeks in June. In order to cover all aspects of the broader national newspaper discourse, the main tabloid and the two biggest quality newspapers – one more leftist-liberal, one more rightist-conservative – were selected (see also Koopmans and Statham 2010; see Table 1).

**Table 1.** Overview of newspapers in the sample.

	Quality conservative	Quality liberal	Tabloid
Finland	Aamulehti	Helsingin Sanomat	Iltasanomat
Germany	Frankfurter Allgemeine	Süddeutsche Zeitung	Bild
UK	The Times	The Guardian	The Sun

The sample includes articles mentioning the domestic parliament in EU affairs or the EP. The data was aggregated at a daily level for each of the nine included newspapers. Data on parliamentary activities was lagged (*t* − 1) to connect it with coverage; we are looking at daily newspapers, which follow the routine of a 24 h news cycle. Other time lags were checked but not found to provide more convincing explanations of coverage. Thus, taking the day within one newspaper as the unit of analysis, observations amount to a total of *N* = 648.^3^ In sum, the analysis measures the influence of parliamentary EU activities of one day on the amount of articles about parliaments in the EU on the next day in nine different newspapers.

Due to an over-dispersion of data, absolute numbers of articles were analysed using a negative binomial regression model. To account for group specific influences, dummy variables were included for countries and outlets. Outlets are included against the background that empirical studies have shown greatest differences between outlets generally (e.g. Boomgaarden et al. 2013). Since the analysis was designed to also detect possible mutual effects of parliamentary communication on news visibility, models always included activities of both levels. In this vein, the activities of one or the other parliamentary level also serve as a control variable.

### Dependent variables

Dependent variables were defined by (1) the absolute number of EP articles and (2) the absolute number of articles on NPs in EU affairs aggregated at the daily level within one newspaper in one country. A very first look at the data revealed that parliaments did not feature in articles together very often. They were therefore not considered as a distinct category in the analysis.

### Independent variables

Independent variables comprise debates, (written and oral) questions and press releases as the most important public activities of parliamentarians and parliaments in terms of their amount and regarding their potential to make parliamentary work visible (see Auel, Eisele, and Kinski 2015 for a similar design). These activities, it is argued, serve the purpose to act as a proxy for parliaments’ efforts to become visible in public and make EU politics transparent for citizens. They are rather easily comparable for all parliaments under study and cover different periods of time; debates and questions are part of official legislative proceedings whereas press releases are not (Shoemaker and Reese 1996, 123). Accordingly, parliamentary debates and questions serve as measures of how political contestants in the parliamentary arena make EU politics visible. Especially debates ‘are vital elements of electoral competition as they provide for a public articulation of societal interests and the discussion of policies, thus informing citizens about complex political issues’ (Auel and Raunio 2014, 13). Debates happen only when parliament is sitting whereas at least written questions can be published continuously.

Press releases present a tool specifically designed to cater to the media’s needs and can be issued on a continuous basis as well. Issuing press releases is seen as an attempt to influence the news agenda (Kiousis et al. 2006, 266) – and thus an active effort by parliament to focus media attention on its activities. In addition, while the levels of public legislative activities are expected to differ greatly, press releases are expected to be more comparable in how parliaments’ employ them. It is argued that they may, therefore, offer a straightforward way to check the robustness of our results and, at the same time, understand the influences caused by the diversity of legislative traditions.

Since press releases often cover what happens within parliament, it was not surprising to find a comparably high correlation between press releases and debates (see Supplementary Table A1 for correlation matrix). For this reason, legislative and media activities are tested in separate models. Data on EP activities was retrieved from the EP’s online archives whereas data on parliamentary activities of NPs in EU affairs was collected in the context of the PACE project and the OPAL project^4^ (Auel, Eisele, and Kinski 2015, 2016; Auel, Rozenberg, and Tacea 2015b). Press releases comprise only those issued by the institution of parliament, not individual party groups or M(E)Ps. Both variables are count data indicating the amount of e.g. debates on one day. Overall, models always compare the EP with one country’s parliament in the same country’s newspapers: e.g. the Bundestag with the EP in German newspapers.

### Control variables

Due to the high salience of the European crisis, a control variable used for contextualising results indicated if articles dealt with financial matters or not. It was coded as 1 = yes if the article focussed on financial issues such as the debt crisis, either entirely or as one topic amongst others.^5^

In a similar vein, we created a dummy variable controlling for the influence of extra-parliamentary crisis events for our robustness checks. This included important dates (=1) as listed by the European Commission.^6^ The original variable was then lagged (*t* − 1) in accordance with the time lag included for parliamentary activities.

## Results

**Figure 1.**
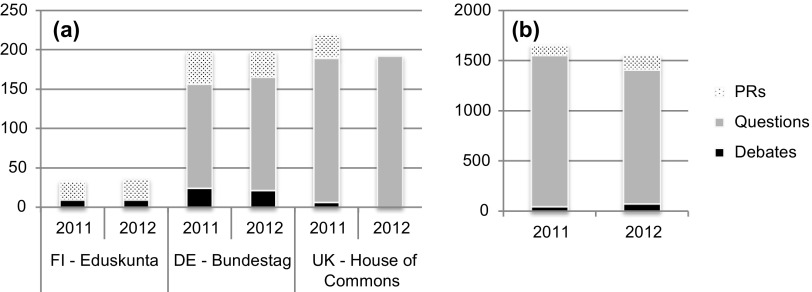
(a) Absolute numbers of EU activities of NPs and (b) absolute numbers of activities of the EP.

Looking at descriptive results concerning activities, it is not surprising that numbers for EP activities are much higher than for each NP respectively since it works exclusively on EU matters. Concerning debates and questions of NPs (see Figure 1(a) and (b); see Supplementary Table A2 for descriptive statistics), the German Bundestag ranks at the middle position, the House of Commons is found to debate EU affairs to a low degree while typically employing the instrument of questioning to a large extent (e.g. Rozenberg et al. 2011). As expected, the Finnish Eduskunta’s publicly visible activities are found to be very low in number (see also Auel and Raunio 2014); press releases show a more homogenous picture across parliaments.

**Figure 2.**
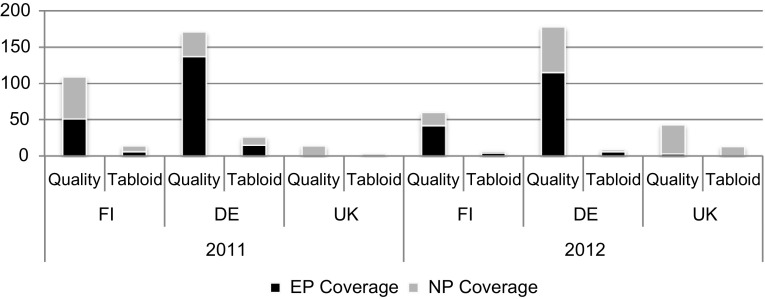
Absolute numbers of articles regarding EP and NP coverage.

Regarding the absolute numbers of newspaper articles (see Figure 2; see Supplementary Table A3 for descriptive statistics), German newspapers are found to produce more news than the Finnish with the UK virtually ignoring the EP as a topic. These country-specific findings are roughly mirrored in the literature analysing EP and other EU-related coverage, too (e.g. Gattermann 2013; Strömbäck et al. 2011). The EP is clearly covered more often than the German Bundestag in EU affairs, while news articles on the Finnish Eduskunta and the EP are shared rather evenly in the period under investigation. In line with the academic state of the art, tabloids cover the topic significantly less than quality outlets (e.g. Boomgaarden et al. 2013).

Turning to the negative binomial regression analysis (see Table 2) results show a positive, significant relationship for EP debates and EP press releases on EP articles and for NP debates on NP articles independently (H_1_). Questions, in contrast, do not play a role at all.

**Table 2.** Effects of legislative and media activities on all articles covering the EP or the NP in EU affairs.

	EP coverage		NP coverage	
	Legislative	Media	Legislative	Media
EP debates	0.0923***	–	0.0910***	–
	(0.0208)		(0.0258)	
EP questions	0.00159	–	0.000144	–
	(0.00215)		(0.00265)	
NP debates	0.134	–	0.394**	–
	(0.0845)		(0.122)	
NP questions	−0.0248	–	0.00380	–
	(0.0311)		(0.0291)	
EP press rel.	–	0.0935***	–	0.0810***
		(0.0161)		(0.0210)
NP press rel.	–	−0.126	–	−0.253
		(0.116)		(0.165)
FI	3.476***	3.547***	0.111	0.179
	(0.592)	(0.589)	(0.203)	(0.198)
DE	4.438***	4.504***	0.314	0.485*
	(0.585)	(0.584)	(0.192)	(0.193)
Tabloid	−1.704***	−1.695***	−0.985***	−0.985***
	(0.199)	(0.197)	(0.193)	(0.194)
Constant	−4.151***	−4.307***	−1.060***	−1.148***
	(0.592)	(0.584)	(0.194)	(0.172)
				
Overdispersion parameter				
Constant	−1.164***	−1.326***	0.00483	0.108
	(0.326)	(0.373)	(0.241)	(0.229)
Observations	630	630	630	630
Pseudo *R*^2^	0.252	0.254	0.054	0.045
AIC	984.2	978.4	1040.9	1046.7

Notes:

Standard errors in parentheses. Note that due to the inclusion of time lags, the number of observations is reduced by 18 (1 day per year and newspaper).

^*^ *p* < 0.05

^**^ *p* < 0.01

^***^ *p* < 0.001.

With regard to mutual influences, the analysis shows a significant positive effect of EP debates and EP press releases on the coverage of NPs in EU affairs while NPs do not affect EP coverage accordingly. This seems to support H_2_ (mutual positive effects), for the EP at least.

Country-specific differences seem more pronounced regarding EP coverage judging from the fact that country dummies have (more) significant effects in EP models. Another interesting result is the difference in the explained variance of models (pseudo *R* ^2^) which is around 20% for EP articles and only 4–5% for NP articles. Thus, while models are comparable in the variables used, they are clearly not when it comes to their explanatory power. This seems to indicate that explanations for NP coverage must also be searched outside the actual parliamentary context. It also highlights the importance for the EP to be publicly visible. More robustness checks were performed which yielded very similar results.^7^

Given the importance of the crisis, we checked if articles discussed financial policies. In NP coverage an overwhelming majority of cases (around 75%) dealt with financial topics. Similar results for a period of four years (2010–2013) have been found in other studies, too (see Auel, Eisele, and Kinski 2015). For EP coverage, in turn, the percentage was much lower, at 28%. Therefore, we included a variable comprising important crisis dates as listed by the European Commission which however did not have any effect and did not change our results. Thus, while the crisis has a great influence, it appears to be country-specific. Parliamentary activities, thus, remain an important explanation for coverage.

Furthermore, ‘given the wide range of variation among national parliaments in the EU, the division of roles with the EP is likely to vary from one state to another’ (Crum and Fossum 2009, 253). We therefore also tested the influence of individual countries in the sample. For that purpose, a jack-knife test was conducted leaving out one country in turn (see Supplementary Tables A7a and A7b for results): Overall, the effect of press releases of NPs and parliamentary questions in general are still insignificant; tabloids still report significantly less in all countries. Press releases of the EP show a stable influence in all regressions on both types of parliamentary coverage. Their positive effect on EP coverage, however, seems to be due to the inclusion of German newspapers which reported most about the EP in terms of absolute numbers.

However, looking into EP press releases more closely, they should be seen as a continuous supply of general EU news: One press release, for example, dealt with a comment by the EP president on Greek bailout politics^8^. In that sense, press releases are EU news mirrored in EP news items that could explain coverage about European politics in general – i.e. of the EP *and* NPs in EU affairs in our case. NP press releases in contrast are found to usually be tailored and limited to the national context.^9^

Regarding debates, EP debates significantly influence EP coverage in all three countries in the sample. The positive influence of EP debates on NP coverage, however, becomes significant only when including Finland. Given the high level of coverage of the Eduskunta compared to the low number of its publicly visible activities to account for it, we looked into Finnish data in more detail finding that coverage here was almost exclusively on financial matters, e.g. relating to actual national parliamentary activities, also discussing parliamentary actors’ extra-parliamentary activities like interviews, for example. Also some EP debates focussed on financial issues and were, for example, dedicated to drafting a position regarding an upcoming Eurosummit (e.g. 13.6.2012^10^). The EP plenary here is then a general stage to discuss salient EU topics and EP positions, but it is the issue itself which is relevant for the media. In sum, external events, and apparently the crisis as an ‘omnipresent’ issue in particular in this period, may act as triggers for both, parliamentary activities and coverage, which could explain the effects in our regression models.

Moreover, the influence of NP debates on NP coverage is significant only when including German newspapers. This seems to be connected to the fact German coverage is the most comprehensive and the Bundestag is also the most active debater by far in our sample.

Eventually, NP debates are found a predictor of EP coverage (*p* < 0.05 when including Finland, *p* < 0.1 when excluding it) which is surprising given that this effect does not occur in our pooled model (see Supplementary Tables A7a and A7b for results).^11^ When estimating results for each country individually (results not shown here), significant effects are only observable for Finland, and not the other two. Thus, the findings of the jack-knife test, which is naturally based on a smaller data-set, might be biased due to the low variance in the British sample.

Turning to the substantive size of effects, the average marginal effects of independent variables for our pooled model is largest for national debates (see Supplementary Figures A1a, A1b, A2a and A2b) but also show the greatest confidence intervals. This is most likely due to the diverse parliamentary traditions and debating cultures included in the comparably small sample that make a more precise prediction difficult. Plotting marginal effects of debates and press releases for EP and NP coverage separately (see Supplementary Figures A3a and A3b) mirrors results of our jack-knife test showing that NP debates seem to, if even minimally, influence EP coverage.

## Conclusion

Summing up, results show a rather robust picture for our pooled model including all three countries. Zooming in on countries individually mirrors results to a great extent although variation is found. All in all, findings imply that structural differences between countries play a role. This concerns legislative traditions with their different approaches to publicity, but also differences in newspaper coverage more generally. In line with similar research (e.g. Gattermann 2013; Strömbäck et al. 2011), coverage in the UK, for example, is much lower than in Germany. Differences are also found between the influence of legislative and media activities at least at the national level where press releases do not play a role in explaining coverage. In addition, it seems that parliamentary plenaries as public fora of political contestation make EU politics as such visible which could contribute to an increase of coverage of both parliamentary levels, although in some countries more than in others.

Given the high share of articles discussing financial policies, the crisis is found a strong moderator of NPs’ coverage. Especially the Finnish Eduskunta did not provide much public activity to report on but was still covered as much as the EP. This may be read as an increase of public controversy in member states regarding a cure for the crisis – maybe even as an expression of the relevance of NPs as guardians of national sovereignty in times of crisis.

Turning to parliaments’ perceived co-operative or competitive relationship as portrayed in newspaper coverage, we searched for mutual effects of activities of one parliamentary level on coverage of the other as an expression of journalists’ underlying perceptions. The literature has shown that EP plenary sessions, albeit weakly, increase EU coverage generally (Boomgaarden et al. 2010); also NP sitting periods and elections can increase EP coverage (Gattermann 2013). Mutual effects, thus, would not simply seem to be happenstance, but we cannot make a final judgement on H_2_ or H_3_ due to the indirect nature of mutual influences found in our results.

Against this background, we conclude that parliaments become visible as independent, complementary branches of parliamentary EU affairs. They seem able to increase their own visibility by being publicly active, which shows that they are perceived as relevant players in EU affairs. Robustness tests suggest that more active parliaments get more coverage, too. They also suggest that country-specific patterns exist. Overall, effect sizes together with the proportionately smaller amount of activities of NPs show that the EP is less successful than its national counterparts in receiving media attention. Thus, broadly speaking, the news value of NPs must be regarded as higher than for the EP, and apparently due to the crisis in particular.

Results will have to be tested on a larger data-set to increase their robustness and allow for generalisation. Findings for EU media coverage have shown rather different tendencies in different EU member states (e.g. Strömbäck et al. 2011). Thus, a different case selection including also Eastern European countries, for example, might reveal patterns diverging from the ones detected here. Moreover, it should be investigated further, if NP’s greater potential of reaching citizens in EU affairs via the news might actually create more room to undermine their legitimacy by looking into the tone of coverage. This would also allow for a better assessment of a perceived potential rivalry or co-operation of parliaments in the eyes of newsmakers. In addition, a more fine-grained understanding of the issues covered and the influence of issue salience is needed. Notwithstanding its shortcomings, however, our explorative study has made a first step towards understanding and explaining the complex interplay of parliaments and communication of and about them in the EU. In that way, it contributes to informing the discussion about the potential of parliaments to help legitimise EU politics and in doing that, extends our knowledge about the EU’s legitimacy and the democratic deficit more generally.

To conclude, given the greater news value of NPs, a division of labour with regard to parliaments input legitimacy in EU affairs is at least imaginable: NPs could tap into their potential to increase input legitimacy regarding their greater impact on newsmakers by debating EU politics publicly – this would support calls for a stronger emphasis of NP’s communication function in EU affairs (Auel 2007). The EP, then, would continue fulfilling controlling functions (Dann 2003) at the EU level. Standards against which it is – negatively – judged as a second order parliament might then have to be adapted to a rather different role that a remote parliament at the supranational level could play for EU citizens at home.

## Disclosure statement

No potential conflict of interest was reported by the author.

## Funding

This work was supported by funding of the Austrian Science Fund (FWF) for the PACE research project (P25062-G16, www.ihs.ac.at/pace/).

## Supplemental data

Supplemental data for this article can be accessed here http://dx.doi.org/10.1080/07036337.2017.1281262.

## Supplementary Material

JEI_Online_Appendix.docx

## Notes

1. We are of course aware of the fact, that cooperating or competing parliaments could also be covered in the same article without considering activities as triggers of coverage. However, a very first look at the data reveals that only a negligible share of 15% deals with both parliaments simultaneously. Therefore they are counted double: Once as EP news and once as NP news. We will therefore not consider this distinct category for hypothesis formulation.

2.
www.ihs.ac.at/pace; see Auel, Eisele, and Kinski (2016).

3. That is 6 (days) multiplied by 6 (weeks) multiplied by 2 (years) multiplied by 3 (countries) multiplied by 3 (newspapers).

4. see also www.ihs.ac.at/pace and www.opal-europe.org

5. Reliability measures for this variable range from 0.84 to 1 for PACE data (Holsti Coefficients tested in country teams (2 coders/country)) and at 0.79 (Krippendorff’s alpha; intra-coder reliability test) for EP news data.

6.
http://ec.europa.eu/economy_finance/crisis/index_en.htm, last accessed on 17 May 2016.

7. To check the robustness of our pooled model, as a first check, articles that covered the EP or the NP as a main topic were used as dependent variable. The ‘main topic’ assessment is based on a qualitative judgement on how important the respective parliament was in the articles in terms its salience for the article under discussion. The question posed was: *Is the EP/NP covered as one of, or the, main topic?* If this was not the case, the topic was defined as being covered in passing while the rest of the article dealt with other news. Reliability measures for this variable for PACE data range from 0.81 to 0.88 (Holsti coefficients tested in county teams) and at 0.68 (Krippendorff’s alpha) for data on EP news. Moreover, two logistic regression models were estimated for each parliamentary level separately which measured the influence of activities on 0 = no EP or NP coverage, and 1 = EP or NP coverage on that day (see Supplementary Tables A7a and A7b for all results). In both tests, results were very similar to results presented here.

8. Press Release of the EP of 23 May 2005: ‘EP president Schulz: act now or risk Europe tearing apart’; accessible at http://www.europarl.europa.eu/news/en/news-room/20120521STO45447/ep-president-schulz-act-now-or-risk-europe-tearing-apart, last accessed on 17 November 2016.

9. This is also a result from qualitative interviews that were conducted in the context of the PACE project with the parliamentary communication services and press offices of party groups.

10. Reference number EP Debate: P7_CRE(2012)06-13 (6); accessible at http://www.europarl.europa.eu/plenary/en/ debates-video.html; last accessed on 17 November 2016.

11. Otherwise we found the almost exact mirror of the jack-knife test results when estimating models for each country individually.
