# Supplementary material for: Complementing, competing, or co-operating? Exploring newspapers’ portrayals of the European Parliament and national parliaments in EU affairs
Source: J Eur Integr. 2017 Jan 31;39(4):435–51. doi: 10.1080/07036337.2017.1281262 (PMC5483338; doi:10.1080/07036337.2017.1281262)

# Appendix

Table A1: Overview of Correlations of Independent Variables (significance level in parentheses)

|  |  | EP | | |  | NP | |
| --- | --- | --- | --- | --- | --- | --- | --- |
|  |  | Debates | Questions | PRs |  | Debates | Questions |
| EP Questions |  | 0.2163 | 1.000 |  |  |  |  |
|  |  | (0.0000) |  |  |  |  |  |
| EP Press Releases |  | 0.4913 | 0.5871 | 1.000 |  |  |  |
|  |  | (0.0000) | (0.0000) |  |  |  |  |
| NP Debates |  | 0.0781 | 0.1135 | 0.2552 |  | 1.000 |  |
|  |  | (0.0500) | (0.0044) | (0.0000) |  |  |  |
| NP Questions |  | -0.0099 | -0.0357 | -0.0026 |  | 0.0042 | 1.000 |
|  |  | (0.8044) | (0.3708) | (0.9487) |  | (0.9153) |  |
| NP Press Releases |  | -0.0210 | 0.1571 | 0.1588 |  | 0.2425 | 0.0601 |
|  |  | (0.5981) | (0.0001) | (0.0001) |  | (0.0000) | (0.1321) |

Table A2: Overview of Descriptive Statistics of Independent Variables Aggregated by Day

|  | Variable | Obs | Mean | Std. Dev. | Min | Max |
| --- | --- | --- | --- | --- | --- | --- |
| European Parliament | Debates | 648 | 1.611111 | 2.753668 | 0 | 10 |
|  | Questions | 648 | 39.93056 | 31.19891 | 0 | 112 |
|  | Press Releases | 648 | 3.527778 | 3.48268 | 0 | 14 |
| National Parliaments’ Activities by Country (N=3x216) | | | | | | |
| FI - Eduskunta | Debates | 216 | .0833333 | .3234983 | 0 | 2 |
|  | Questions | 216 | 0 | 0 | 0 | 0 |
|  | Press Releases | 216 | .2361111 | .4256778 | 0 | 1 |
| DE - Bundestag | Debates | 216 | .2083333 | .7269721 | 0 | 4 |
|  | Questions | 216 | 1.277778 | 2.756247 | 0 | 14 |
|  | Press Releases | 216 | .3472222 | .60601 | 0 | 2 |
| UK - House of  Commons | Debates | 216 | .0277778 | .1647173 | 0 | 1 |
|  | Questions | 216 | 1.736111 | 3.892723 | 0 | 28 |
|  | Press Releases | 216 | .1388889 | .4194866 | 0 | 2 |

Table A3: Overview of Descriptive Statistics of Dependent Variables Aggregated by Day

| Variable | Obs | Mean | Std. Dev. | Min | Max |
| --- | --- | --- | --- | --- | --- |
| EP Articles | 648 | .5848765 | 1.189425 | 0 | 8 |
| NP Articles | 648 | .4135802 | .8515469 | 0 | 6 |

Table A4: Overview of Dependent Variables Used for Robustness Checks

|  | Obs | Mean | Std. Dev. | Min | Max | No. of Art. Main Topic | All Articles |
| --- | --- | --- | --- | --- | --- | --- | --- |
| *Neg. Binomial Regression Models* | | | | | | | |
| EP Main Topic | 648 | .1790123 | .5821064 | 0 | 5 | 116 | 379 |
| NP Main Topic | 648 | .1635802 | .4695693 | 0 | 3 | 106 | 268 |
| *Logit Regression Models* | | | | | | | |
| EP Logit | 648 | .3040123 | .4603432 | 0 | 1 |  |  |
| NP Logit | 648 | .2731481 | .4459205 | 0 | 1 |  |  |

Table A5: Negative Binomial Regression: Effects of Legislative and Media Activities on Articles Covering EP or NPs as Main Topic

|  |  | EP | |  | NP | |
| --- | --- | --- | --- | --- | --- | --- |
|  |  | Legislative | Media |  | Legislative | Media |
|  |  |  |  |  |  |  |
| EP Debates |  | 0.117** | -- |  | 0.0904* | -- |
|  |  | (0.0370) |  |  | (0.0356) |  |
|  |  |  |  |  |  |  |
| EP Questions |  | 0.00597 | -- |  | 0.00239 | -- |
|  |  | (0.00384) |  |  | (0.00387) |  |
|  |  |  |  |  |  |  |
| NP Debates |  | 0.162 | -- |  | 0.565*** | -- |
|  |  | (0.137) |  |  | (0.139) |  |
|  |  |  |  |  |  |  |
| NP Questions |  | -0.156 | -- |  | 0.0318 | -- |
|  |  | (0.0928) |  |  | (0.0388) |  |
|  |  |  |  |  |  |  |
| EP Press Rel. |  | -- | 0.147*** |  | -- | 0.0956** |
|  |  |  | (0.0301) |  |  | (0.0291) |
|  |  |  |  |  |  |  |
| NP Press Rel. |  | -- | -0.159 |  | -- | -0.357 |
|  |  |  | (0.214) |  |  | (0.249) |
|  |  |  |  |  |  |  |
| Dummy FI |  | 17.65 | 17.05 |  | 0.933** | 0.964** |
|  |  | (1437.3) | (937.1) |  | (0.311) | (0.304) |
|  |  |  |  |  |  |  |
| Dummy DE |  | 18.85 | 18.16 |  | 0.503 | 0.787* |
|  |  | (1437.3) | (937.1) |  | (0.318) | (0.315) |
|  |  |  |  |  |  |  |
| Dummy Tabloid |  | -1.124*** | -1.072*** |  | -0.865** | -0.852** |
|  |  | (0.302) | (0.300) |  | (0.278) | (0.281) |
|  |  |  |  |  |  |  |
| Constant |  | -19.97 | -19.44 |  | -2.600*** | -2.575*** |
|  |  | (1437.3) | (937.1) |  | (0.326) | (0.288) |
| Overdispersion Parameter | | |  |  |  |  |
| Constant |  | 0.0608 | 0.0417 |  | -0.155 | 0.256 |
|  |  | (0.392) | (0.404) |  | (0.548) | (0.435) |
| Observations |  | 630 | 630 |  | 630 | 630 |
| Pseudo *R*^2^ |  | 0.205 | 0.200 |  | 0.078 | 0.054 |

Standard errors in parentheses; * p<0.05, ** p<0.01, *** p<0.001; note that due to the inclusion of time lags, the number of observations is reduced by 18: 1 day per year and newspaper.

Table A6: Logistic Regression: Effects of Activities on Days in a Newspaper with Coverage (=1)

|  |  | EP | |  | NP | |
| --- | --- | --- | --- | --- | --- | --- |
|  |  | Legislative | Media |  | Legislative | Media |
|  |  |  |  |  |  |  |
| EP Debates |  | 0.136^**^ | -- |  | 0.0906^**^ | -- |
|  |  | (0.0424) |  |  | (0.0327) |  |
|  |  |  |  |  |  |  |
| EP Questions |  | -0.00150 | -- |  | -0.00326 | -- |
|  |  | (0.00377) |  |  | (0.00318) |  |
|  |  |  |  |  |  |  |
| NP Debates |  | 0.441 | -- |  | 0.678^**^ | -- |
|  |  | (0.240) |  |  | (0.209) |  |
|  |  |  |  |  |  |  |
| NP Questions |  | 0.00490 | -- |  | 0.0239 | -- |
|  |  | (0.0538) |  |  | (0.0308) |  |
|  |  |  |  |  |  |  |
| EP Press Rel. |  | -- | 0.0979^**^ |  | -- | 0.0738^**^ |
|  |  |  | (0.0328) |  |  | (0.0258) |
|  |  |  |  |  |  |  |
| NP Press Rel. |  | -- | 0.101 |  | -- | -0.306 |
|  |  |  | (0.215) |  |  | (0.193) |
|  |  |  |  |  |  |  |
| FI |  | 3.717^***^ | 3.703^***^ |  | -0.158 | -0.131 |
|  |  | (0.620) | (0.608) |  | (0.239) | (0.231) |
|  |  |  |  |  |  |  |
| DE |  | 5.036^***^ | 5.051^***^ |  | 0.173 | 0.352 |
|  |  | (0.617) | (0.615) |  | (0.226) | (0.223) |
|  |  |  |  |  |  |  |
| TAB |  | -2.264^***^ | -2.221^***^ |  | -0.950^***^ | -0.925^***^ |
|  |  | (0.281) | (0.276) |  | (0.218) | (0.214) |
|  |  |  |  |  |  |  |
| Constant |  | -4.143^***^ | -4.311^***^ |  | -0.840^***^ | -0.987^***^ |
|  |  | (0.626) | (0.606) |  | (0.221) | (0.194) |
| Observations |  | 630 | 630 |  | 630 | 630 |
| Pseudo *R*^2^ |  | 0.378 | 0.370 |  | 0.062 | 0.045 |

Standard errors in parentheses; * p<0.05, ** p<0.01, *** p<0.001; note that due to the inclusion of time lags, the number of observations is reduced by 18: 1 day per year and newspaper.

Table A7a: Jack-knife Test: Effects of Press Releases on Coverage

|  |  | (1) | (2) | (3) |  | (1) | (2) | (3) |
| --- | --- | --- | --- | --- | --- | --- | --- | --- |
|  |  | No. Articles EP | | |  | No. Articles NP | | |
|  |  |  |  |  |  |  |  |  |
| EP Press Rel. |  | 0.0962*** | 0.0349 | 0.113*** |  | 0.0949*** | 0.0805** | 0.0645** |
|  |  | (0.0180) | (0.0348) | (0.0268) |  | (0.0259) | (0.0268) | (0.0250) |
|  |  |  |  |  |  |  |  |  |
| NP Press Rel. |  | -0.0196 | 0.440 | 0.191 |  | -0.128 | -0.252 | -0.223 |
|  |  | (0.129) | (0.288) | (0.186) |  | (0.188) | (0.246) | (0.184) |
|  |  |  |  |  |  |  |  |  |
| Tabloid |  | -1.693*** | -1.580*** | -1.787*** |  | -1.211*** | -0.858*** | -0.910*** |
|  |  | (0.205) | (0.369) | (0.272) |  | (0.248) | (0.244) | (0.224) |
|  |  |  |  |  |  |  |  |  |
| Constant |  | -0.214# | -1.316*** | -0.662*** |  | -0.863*** | -1.078*** | -0.831*** |
|  |  | (0.110) | (0.198) | (0.157) |  | (0.158) | (0.163) | (0.144) |
| **Country excluded:** |  | **UK** | **DE** | **FI** |  | **UK** | **DE** | **FI** |
| Overdispersion Param. |  |  |  |  |  |  |  |  |
| Constant |  | -0.591* | 0.780** | 0.578** |  | 0.294 | 0.238 | -0.102 |
|  |  | (0.236) | (0.302) | (0.188) |  | (0.244) | (0.286) | (0.305) |
| Observations |  | 420 | 420 | 420 |  | 420 | 420 | 420 |
| Pseudo *R*^2^ |  | 0.097 | 0.050 | 0.074 |  | 0.051 | 0.033 | 0.034 |
| *AIC* |  | 994.4 | 498.3 | 831.8 |  | 732.1 | 652.1 | 717.6 |

Standard errors in parentheses; # p<0.10, * p<0.05, ** p<0.01, *** p<0.001

Table A7b: Jack-knife Test: Effects of Debates and Questions on Coverage

|  |  | (1) | (2) | (3) |  | (1) | (2) | (3) |
| --- | --- | --- | --- | --- | --- | --- | --- | --- |
|  |  | No. Articles EP | | |  | No. Articles NP | | |
|  |  |  |  |  |  |  |  |  |
| EP Debates |  | 0.0978*** | 0.114** | 0.0817* |  | 0.118*** | 0.110*** | 0.0476 |
|  |  | (0.0228) | (0.0419) | (0.0332) |  | (0.0322) | (0.0334) | (0.0308) |
|  |  |  |  |  |  |  |  |  |
| EP Questions |  | 0.00269 | -0.00349 | 0.00411 |  | 0.000563 | 0.0000441 | -0.000194 |
|  |  | (0.00232) | (0.00430) | (0.00323) |  | (0.00334) | (0.00348) | (0.00302) |
|  |  |  |  |  |  |  |  |  |
| NP Debates |  | 0.201* | 0.818* | 0.281# |  | 0.403** | 0.489 | 0.426*** |
|  |  | (0.0951) | (0.352) | (0.144) |  | (0.128) | (0.301) | (0.119) |
|  |  |  |  |  |  |  |  |  |
| NP Questions |  | 0.0408 | -14.21 | -0.0489 |  | 0.0476 | -0.0189 | -0.00263 |
|  |  | (0.0321) | (492.9) | (0.0343) |  | (0.0447) | (0.0395) | (0.0276) |
|  |  |  |  |  |  |  |  |  |
| Tabloid |  | -1.704*** | -1.641*** | -1.788*** |  | -1.219*** | -0.853*** | -0.904*** |
|  |  | (0.205) | (0.373) | (0.271) |  | (0.247) | (0.243) | (0.222) |
|  |  |  |  |  |  |  |  |  |
| Constant |  | -0.200 | -1.027*** | -0.465* |  | -0.911*** | -1.056*** | -0.797*** |
|  |  | (0.131) | (0.210) | (0.185) |  | (0.184) | (0.187) | (0.169) |
| **Country excluded:** |  | **UK** | **DE** | **FI** |  | **UK** | **DE** | **FI** |
| Overdispersion Param. |  |  |  |  |  |  |  |  |
| Constant |  | -0.594* | 0.223 | 0.546** |  | 0.131 | 0.138 | -0.275 |
|  |  | (0.236) | (0.355) | (0.193) |  | (0.263) | (0.301) | (0.337) |
| Observations |  | 420 | 420 | 420 |  | 420 | 420 | 420 |
| Pseudo *R*^2^ |  | 0.099 | 0.138 | 0.074 |  | 0.068 | 0.043 | 0.046 |
| *AIC* |  | 997.1 | 456.8 | 836.0 |  | 723.1 | 649.6 | 712.6 |

Standard errors in parentheses; # p<0.10, * p<0.05, ** p<0.01, *** p<0.001

Figure A1a: Average Marginal Effects Plots after negative binomial regression (see table 2): Effect of Press Releases on EP Coverage and NP Coverage:

Marginal Effects of Press Releases on *EP Coverage* (at means)


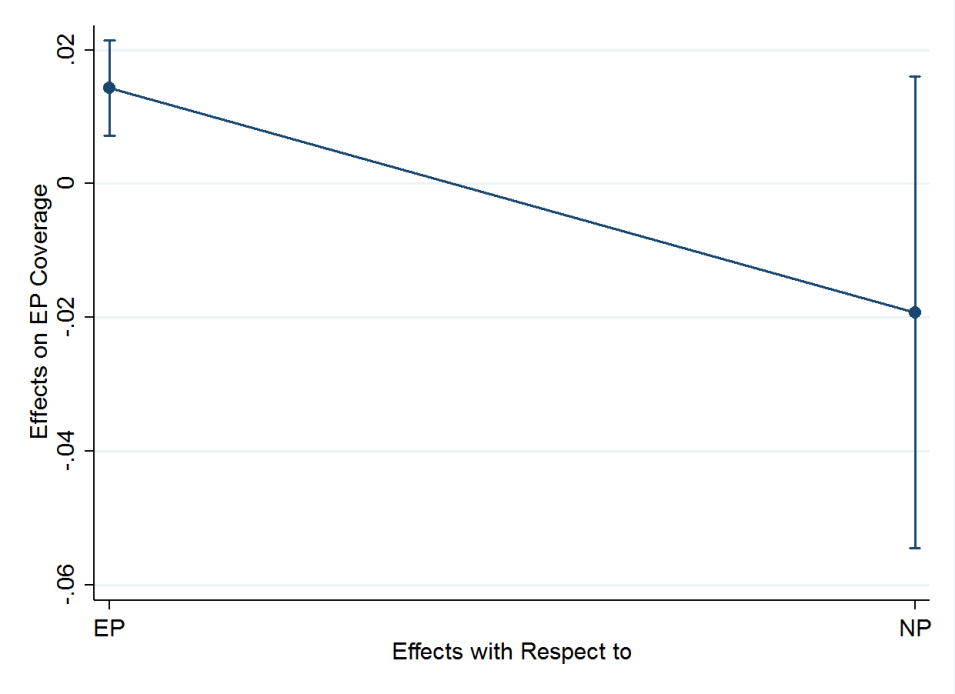


Figure A1b: Average Marginal Effects Plots after negative binomial regression (see table 2): Effect of Press Releases on EP Coverage and NP Coverage:

Marginal Effects of Press Releases on *NP Coverage* (at means)


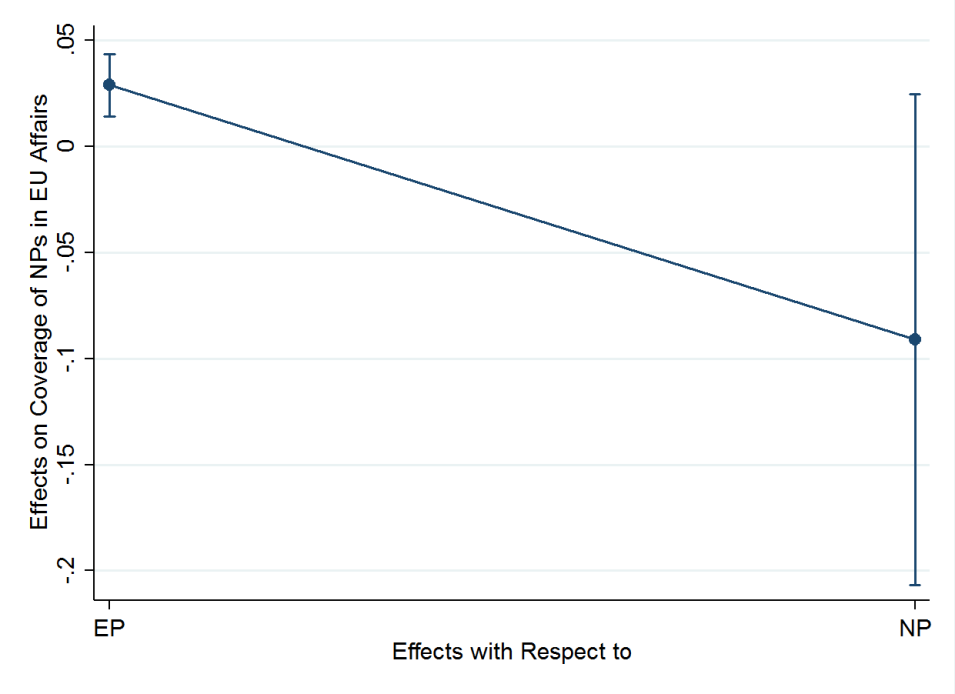


Figure A2a: Average Marginal Effects Plots after negative binomial regression (see table 2): Effect of Press Releases on EP Coverage and NP Coverage: Marginal Effects of Debates and Questions on *EP Coverage* (at means)


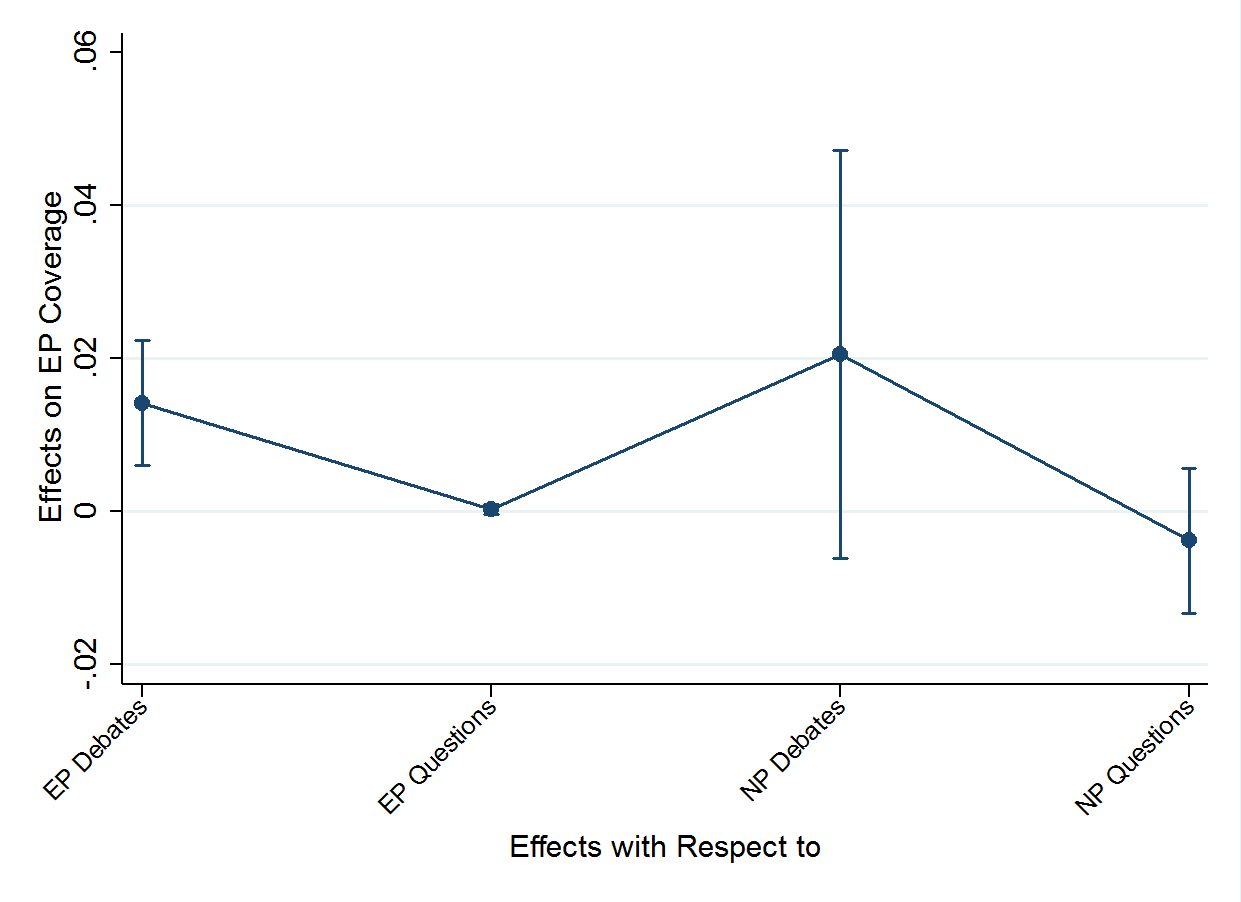


Figure A2b: Average Marginal Effects Plots after negative binomial regression (see table 2): Effect of Press Releases on EP Coverage and NP Coverage: Marginal Effects of Debates and Questions on *NP Coverage* (at means)


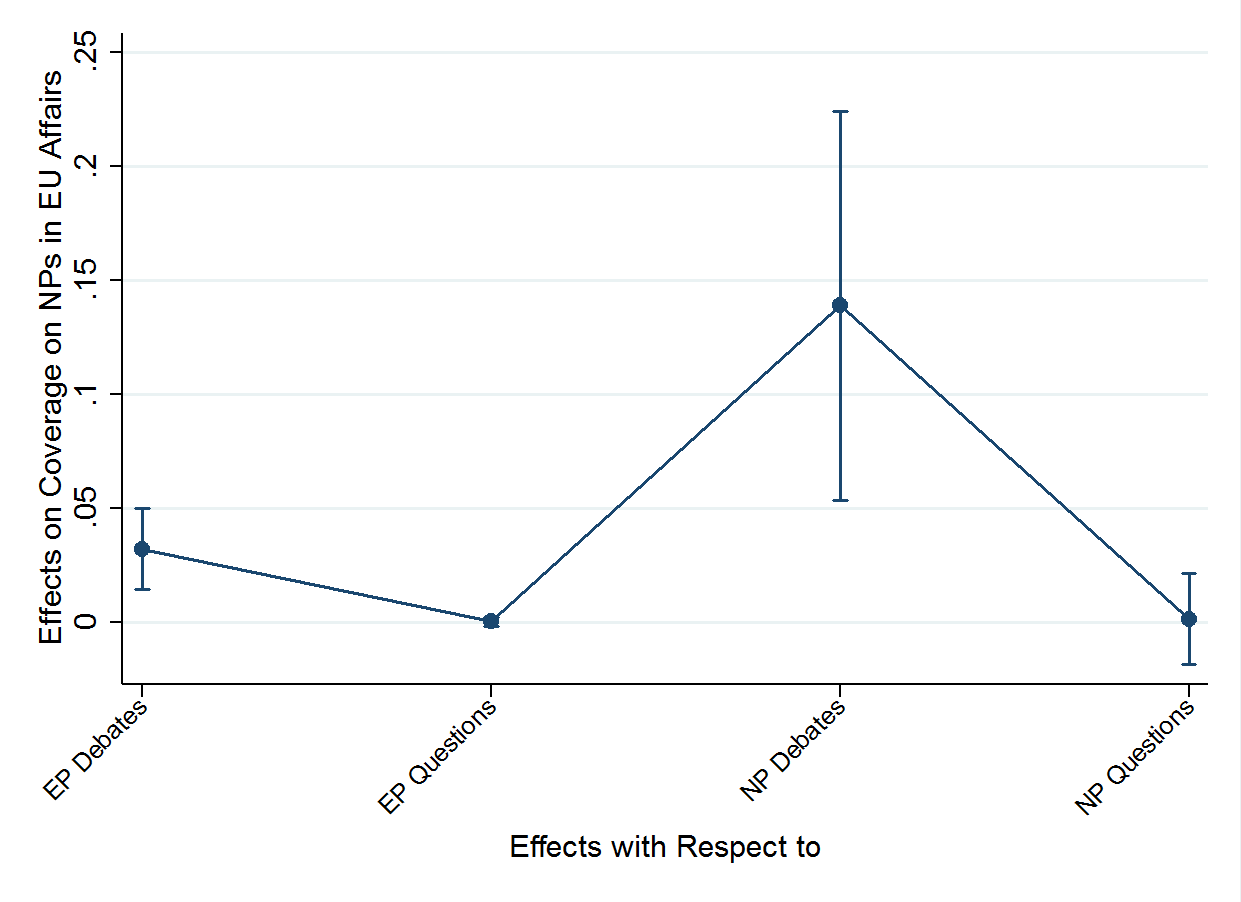


Figure A3a: Marginal Effects Plots after negative binomial regression (see table 2): Marginal Effects of Debates on EP Coverage and NP Coverage


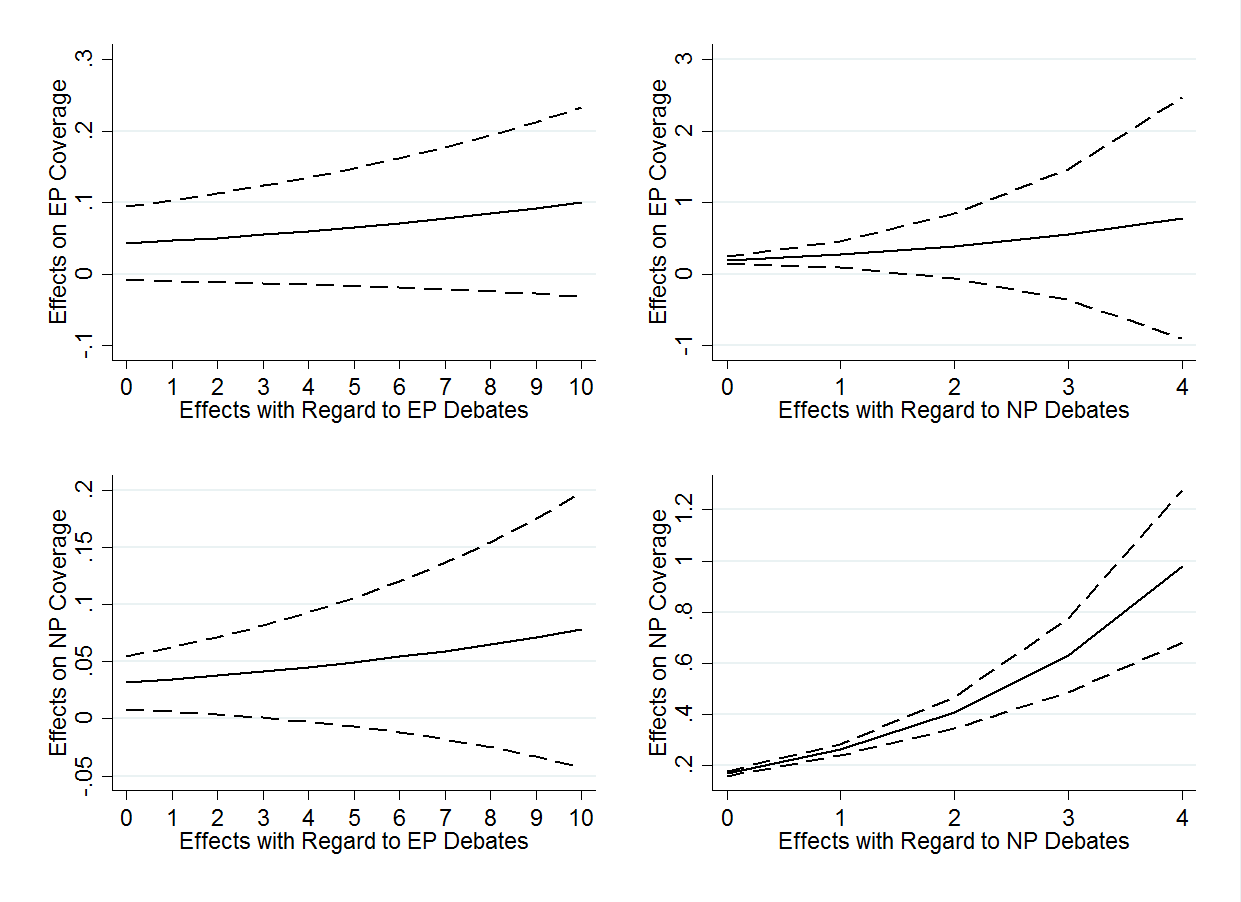


Figure A3b: Marginal Effects Plots after negative binomial regression (see table 2): Marginal Effects of Press Releases on EP Coverage and NP Coverage


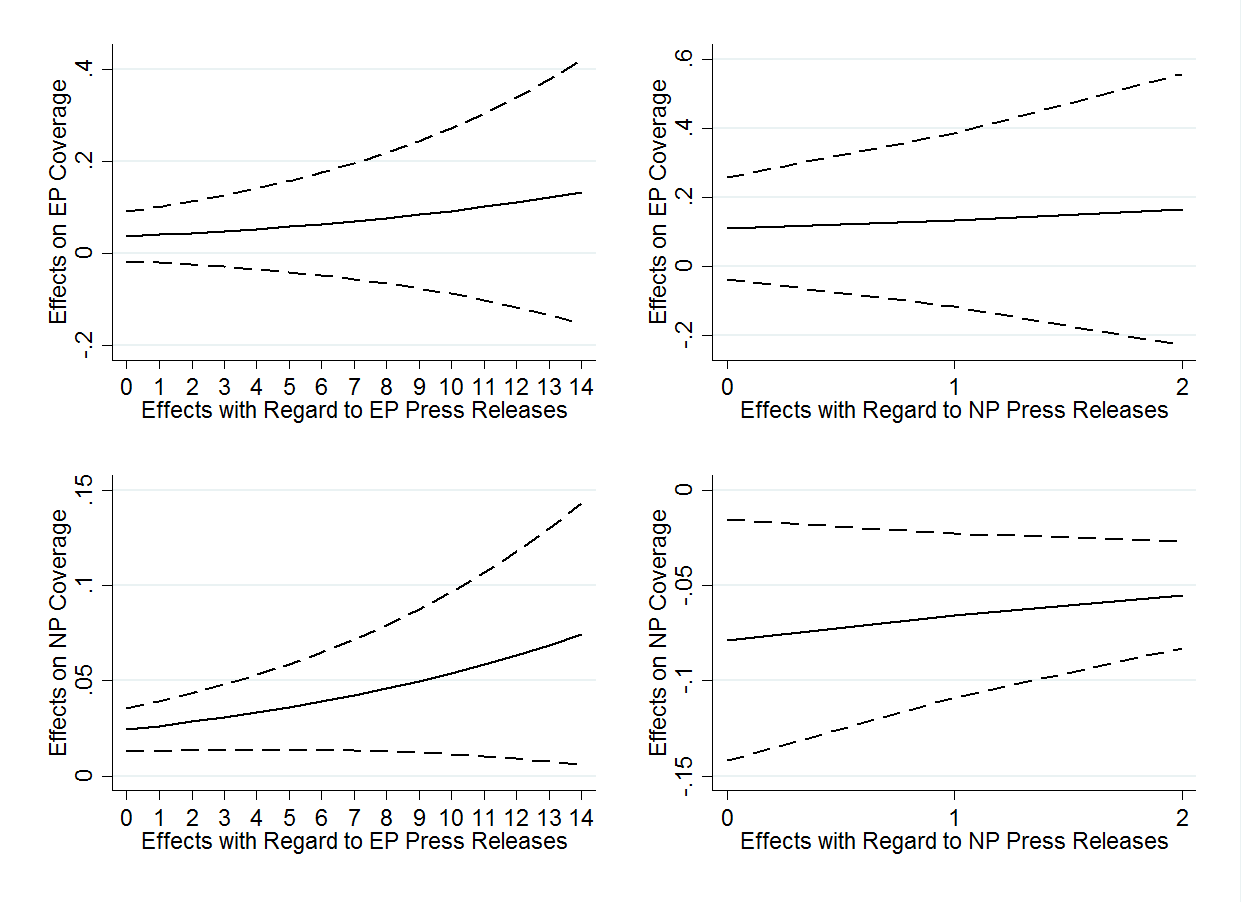

Supplement: JEI_Online_Appendix.docx [file geui_a_1281262_sm4686.docx]
